# Research on a Novel MEMS Sensor for Spatial DC Electric Field Measurements in an Ion Flows Field

**DOI:** 10.3390/s18061740

**Published:** 2018-05-28

**Authors:** Ya Mou, Zhanqing Yu, Kaitian Huang, Qing Ma, Rong Zeng, Zheyao Wang

**Affiliations:** 1 State Key Lab of Power Systems, Department of Electrical Engineering, Tsinghua University, Beijing 100084, China; mouy17@mails.tsinghua.edu.cn; 2 Electric Power Research Institute, China Southern Power Grid, Guangzhou 510000, China; andykaitian@163.com; 3 Department of Microelectronics and Nanoelectronics, Tsinghua University, Beijing 100084, China; maqing402@163.com (Q.M.); z.wang@mail.tsinghua.edu.cn (Z.W.)

**Keywords:** ion flow, electric field sensor, MEMS, potential independent, differential structure

## Abstract

Thus far, despite the development of electric field sensors (EFSs) such as field mills, optoelectronic EFSs and microelectromechanical system (MEMS)-based EFSs, no sensor can accurately measure an electric field in space due to the existence of space charge and the influence of charge attachment. To measure a spatial synthetic electric field in an ion flow field, a double potential independent differential EFS based on MEMS is proposed. Compared with other EFSs, this method has the advantages of independent potential (without grounding) and the ability to support the measurement of the synthetic ion flow electric field in space. First, to analyse the charge distribution after the sensor is involved exposed to an electric field, a simulation model was constructed. Then, given the redistribution of the spatial electric field in space and the influence of the surface charge on the sensor, the quantitative relationship between the electric field to be measured and that measured by the proposed sensor was obtained. To improve the performance of the EFS, a set of synthetic field strength sensor calibration systems that consider spatial ion flow injection was established. Furthermore, the parameter *λ*, which is related to the relative position of the differential chips, was determined. Finally, a series of comparative experiments indicated that the differential EFS highlighted in the present study exhibits good linearity and accuracy.

## 1. Introduction

With the industrial developments and improvement in living standards, the demand for electricity has increased, and high-voltage, direct current (HVDC) transmission lines have been widely developed worldwide because of their advantages of long transmission distance, large transport capacity and low cost. However,-the increased voltage level introduces problems related to the electromagnetic environment, such as an increase in the synthetic electric field, audible noise, radio interference, and loss of ion flow [1].

Corona discharge occurs on the surface of an HVDC transmission line when the electric field strength exceeds the air breakdown field strength [2,3]. When a corona appears near HVDC transmission lines, charged particles with an opposite polarity to that of the transmission line move towards the electrode under the action of the electric field, absorbing or losing electrons on the transmission line surface, thereby restoring electrical neutrality and resulting in a corona current. Additionally, the same polar charged particles are excluded from the transmission line and move along the electric field line, gradually spreading to the space surrounding the transmission line and finally pouring into the earth. The strength of synthetic field is associated not only with the operating voltage and mode of the HVDC transmission line but also with the corona discharge strength.

For a stronger smart power grid, a broadband, full-time coverage, high-sensitivity sensing system is an effective strategy against emergencies in a power system, such as lightning, operating trouble, and insulation failures. Among the four parameters of ion flow, audible noise, synthetic electric field and radio interference, the synthetic electric field closely interacts with or otherwise affects the insulation of a material [1], the operating state of the power system and biological health. To date, studies on synthetic electric field problems in HVDC transmission lines have mainly been conducted using ionized-field numerical calculations and measuring methods or devices. Using ionized-field numerical calculations, many researchers have proposed algorithms to calculate the ionized-field distribution under HVDC lines [4], such as the finite element method [5,6]. However, the drawbacks of complexity and incompleteness of the calculation methods—specifically, that no single algorithm can consider all aspects simultaneously and the numerical calculation method may not guarantee the absolute accurateness of results—have prompted the development of sensors or devices with good performance for electric field measurements.

Over the past decades, field mills were most commonly used for measuring ionized fields because of their stability and linearity [7,8]. Field mills are primarily composed of shielding electrodes, sensing electrodes, motors for providing rotary power and signal processing circuits. The motor periodically rotates the shielding electrodes, resulting in periodic exposure of the sensing electrodes to the electric field; consequently, different amounts of charge appear on the surface of the sensing electrodes according to the electrostatic induction law. Notably, the shielding electrodes must be grounded to exclude the effects of ion flow, suggesting that this solution is inappropriate in space. Furthermore, field mills are so bulky (generally 8 cm × 10 cm × 10 cm) that they distort the original electric field. 

Recently, smaller-sized devices based on the Pockels effect [2,3], electro-optical Kerr effect [9], microelectronic mechanical system (MEMS) technology [10] or the piezoelectric effect [11] have been developed to measure electric fields. Optical EFSs have the advantages of a fast transient response and a wide measuring range and are used to measure AC electric fields, especially transient electric fields. Reasonably, optical EFSs require a light source, optical fibre, and optical detector monitor, resulting in a bulky the measuring system and increasing costs of the system. Because they are lightweight, small, easily integrated and easily mass produced, MEMS-based sensors are popular with researchers at home and abroad. Thus far, the application of several structures based on MEMS technology in DC EFSs has been proposed; including double vertical vibration [12,13], double parallel movement [14] and single parallel movement [15].

Recently, a serious of new type EFSs have been proposed. Chu proposed a new type based on torsional resonance [16], and Andò designed a ferroelectric-capacitor-based quasistatic EFS [17]. Kainz et al. used the AC electric field to generate the driving force which makes the optical shutter of the MEMS structure periodically shielding the light and changes the light flux received by the photodetector, so as to realize the detection of the low-frequency AC electric field and DC electric field [18]. However, this method still requires a light source, which has the imperfections with high-cost and inconvenience. In addition, when measuring the vertical electric field, the seismic mass moves vertically, affecting the measuring accuracy due to the effect of gravity. Furthermore, it cannot be used for the measurement in ion flow fields without considering ion attachment problem. Comparisons of different EFS are listed in Table 1. However, the measuring result cannot be assumed to be equal to the original value due to the redistribution of the charged particles and electric field distortion when the sensor is placed in an ion flow field [16,17,18,19]. Thus far, no effective method has been developed to measure the space synthetic field in an ionized field. In the ion flow field, the ion flow moves onto the top shell of the sensor, charges the top plates and generates a zero-charge area around the sensor. Thus, a MEMS sensor in an ion flow field senses not only the original electric field but also the changes in the field caused by distortion around the sensor, which makes measurements unreliable. Thus, an understanding of the charging process of the charged articles and the relationship between the original electric field and the measured electric field is necessary to accurately measure the space synthetic field in an ionized field.

To measure the space synthetic electric field in the ion flow, we designed a double-potential independent EFS based on MEMS technology. Compared with other EFSs, this technology has the advantages of small volume, independent potential (without grounding), and the ability to support the measurement of the synthetic electric field in space. As a first step in the present study, we designed an electrostatic comb-drive and capacitive induction EFS and analysed the measurement principles. We then constructed a multi-physical simulation model to identify the relationship between the measured value and the electric field to be measured. Based on measurements in the electrostatic field, we deduced the relationship between the output voltage and the electric field. The calibration process was subsequently analysed in detail to obtain the parameter *λ*. To verify the accuracy of this measurement, we conducted a series of experiments and compared the measured results with the calculated results, which showed that the proposed sensor exhibits good linearity and high accuracy. 

## 2. Principle of the Sensor

### 2.1. Principle of the Sensor

MEMS technology is widely used in consumer electronics (such as accelerometers, gyroscopes, inertial devices, and MEMS microphones) for its lightweight, low-cost, low-power-consumption and easy batch production [9]. With the continuous maturation of processing technology, MEMS technology has been gradually applied in the field of medical and communication systems [19,20]. Specifically, as a wider frequency band is supported by smartphones, radio-frequency (RF) MEMS devices have emerged as promising critical devices in the 5G communication revolution [21]. Moreover, MEMS technology has been used in EFSs since 1992 [22].

**Figure 1:**
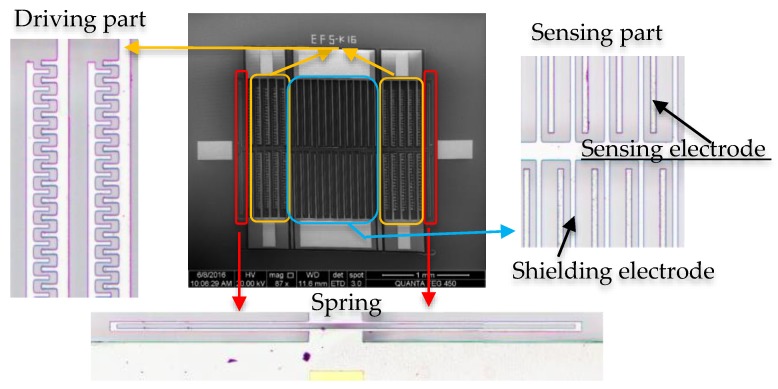
The structure of an electric field sensor based on MEMS technology.

As is shown in Figure 1, EFSs based on MEMS processing technology consist of a driving part, a sensing part and a spring part [23]. Compared with thermal driving, the electrostatic comb-drive adopted in the present design has the advantage of lower power consumption [23]. The application of a driving voltage can provide the driving force for shielding electrode movement. The spring part functions as an amplifier for the displacement of the shielding electrode, which is beneficial for detection. The sensing part contains a shielding electrode, which is removable, and the sensing electrode, which is fixed by an anchor.

**Figure 2:**
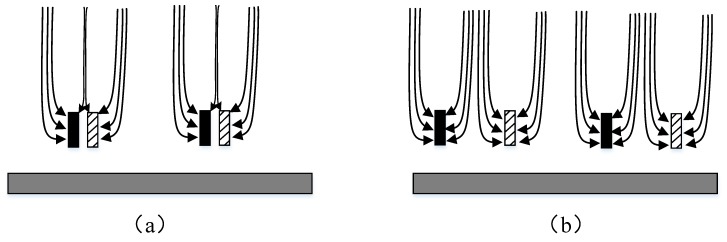
Schematic of electric field shielding: (**a**) shows the situation in which the shielding electrode is near the sensing electrode, and (**b**) shows the situation in which the shielding electrode is far from the sensing electrode.

A capacitive induction structure is used to detect the electric field (Figure 2). The solid black rectangle and shadow rectangle in Figure 2 represent the shielding electrode and the sensing electrode, respectively, and the grey rectangle represents the substrate of the EFS. When the shielding electrode is near the sensing electrode, the shielding electrode will shield the electric field in the region around the sensing electrode, thus reducing the inducted charge. When the shielding electrode is far from the inducted electrode, the inducted charge on the inducted electrode will increase. If the inducted charge on the sensing electrode is detectable, then the electric field can be determined.

### 2.2. Driving Part

**Figure 3:**
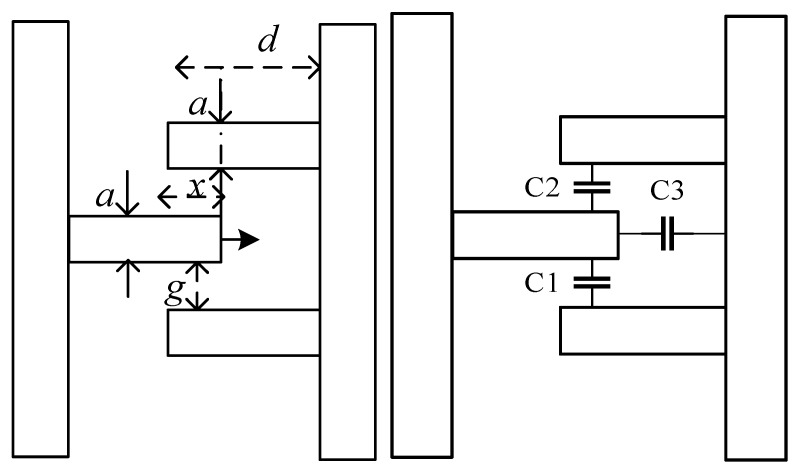
Schematic of the driving part.

Compared with thermal driving, electrostatic driving has the following characteristics: the driving current is small, approaching zero; as a consequence the resistance loss is extremely small; and the structure of the EFS is sufficiently sound to not be broken by heat. A schematic of the electrostatic driving structure is shown in Figure 3.

When a driving voltage is applied at both ends of the drive electrode, an inducted charge will be produced at both ends of the electrode. Electrostatic attraction is induced under the joint action of a positive inducted charge on the positive electrode and a negative inducted charge on the negative electrode.

The left electrodes are fixed by an anchor, and the right electrodes are removable. The total driving capacitance *C* is:

(1) $$C=n(C_{1}+C_{2}+C_{3})=n[\frac{\varepsilon ah}{d-x}+\frac{2\varepsilon xh}{g}]$$

Thus, the driving force *F_x_* is:

(2) $$F_{x}=\frac{\partial W}{\partial x}=\frac{n\varepsilon ahU^{2}}{2{\left(d-x\right)}^{2}}+\frac{n\varepsilon hU^{2}}{g}$$

### 2.3. Sensing Part

When a conductor is placed in an electric field, an inducted charge is generated on the surface of the conductor. In the present EFS, multiple capacitors act as sensing structures.

The capacitance of the sensing part is:

(3) $$C_{s}=n_{s}\varepsilon_{0}\frac{A_{s}}{D}$$

where *n_s_* represents the number of the sensing capacitors, *A_s_* represents the effective area of the sensing electrode, and *D* represents the distance between the sensing electrode and the ground.

Thus, the inducted charge is:

(4) $$Q=C_{s}\cdot U_{s}=n_{s}\varepsilon_{0}\frac{A_{s}}{D}\cdot E\cdot D=n_{s}\varepsilon_{0}A_{s}\cdot E$$

As a result, the inducted current is:

(5) $$i=\frac{\partial Q}{\partial t}=\varepsilon_{0}n_{s}E\frac{dA_{s}}{dt}$$

and the effective induced current is:

$$I=2\omega \varepsilon_{0}n_{s}EA_{s}$$

### 2.4. Design and Fabrication

The fabrication feasibility and performance of the sensor are the main aspects that should be considered. To achieve high linearity and strong output signals, a simulation process is needed. The size of each part of the sensor can be is determined by parameter scanning analysis, as shown in Table 2.

The EFS is fabricated on a silicon-on insulator (SOI) wafer with a resistivity of 0.01 Ω∙cm. First, a SiO_2_ layer as an electrical isolation layer, is deposited on the device layer by a plasma-enhanced chemical vapour deposition (PECVD) process. Then, after etching the SiO_2_ layer by a reactive ion etching (RIE) process to form contact holes, an Al layer is sputtered on the holes to form pad. After that, deep reactive ion etching (DRIE) is used to etch the device layer to form the main structure of the EFS. A polyimide layer is used to protect the structure from destroyed in the subsequent process. Then, the Si substrate is etched by DRIE from the back and the buried oxide layer is etched from the back with RIE. Finally, the polyimide layer is released, forming a suspended structure.

## 3. Modelling of Ion Flow

### 3.1. Description of the Sensor

The MEMS EFS is a few cubic centimetre device in size. The packaging of the device is composed of a hollow metal cylinder with an insulating material on the side surfaces. If the top plane of the package is not a conductor, then the dielectric material will accumulate charge and subsequently become polarized, generating an uncertain electric field, resulting in the measurement meaningless. When considering above reasons, the material of top plate of the EFS is determined as metal.

**Figure 4:**
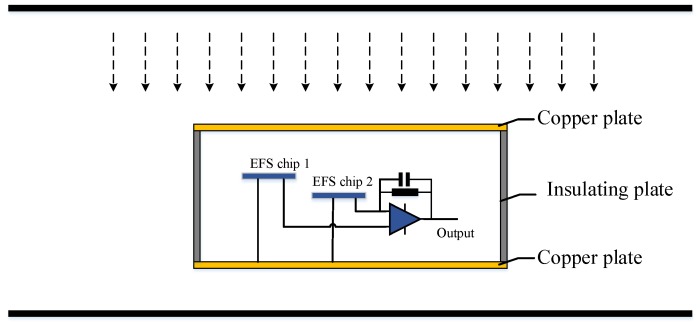
MEMS electric sensor in ion flow field.

The lower metal plate provides a stable grounding potential for the sensor chips and signal processing circuits. The side is made of an insulating material, maintaining a stable potential difference between the top and bottom plate and thus providing a stable internal electric field. As shown in Figure 4, the material of the upper and lower plates is copper and the material of the sidewall is SiO_2_.

In Figure 4, the black parallel lines represent the transmission line and ground. The device in the middle is a measurement sensor composed of two EFS chips and a signal processing circuit.

### 3.2. Analysis of Space Electric Field Injected by Sensor

The EFS was placed in the space below the transmission line to measure the space synthetic electric field. Additionally, the size of the EFS covers tens of cubic centimetres.

In the ion flow field, due to the principle of conservation of charge and current, charge accumulates on the middle interface because of the difference between the conductivity and the dielectric constants of the two sides. If the time at which the first charged particle reaches the metal interface is defined as *t*_0_ and the time when the surface synthetic electric field is zero is defined as *t*_1_, then a transient process current flows through the middle interface from *t*_0_ to *t*_1_.

The zero-field model was used to analyse the charge accumulation. That is, charged particles continuously reach the metal interface to neutralize the charges of opposite polarity on the metal plate until the surface synthetic electric field on the middle interface reaches zero. According to the principle of conservation of electric charge, when current densities from the two sides are *J*_1_ and *J*_2_, the following equation is used:

−*J*_1_S + *J*_2_S = 0 (6)

The differential form of Ohm’s theorem is:

*J* = *δE* (7)

Thus:

*δ*_1_*E*_1_ = *δ*_2_*E*_2_ = *J* (8)

The Gauss theorem is:

*δ*_2_*E*_2_ − *δ*_1_*E*_1_ = *δ*_0_ (9)

Thus, the accumulated charge density on the middle interface is:

(10) $$\delta_{0}=(\frac{\varepsilon_{2}}{\delta_{2}}-\frac{\varepsilon_{1}}{\delta_{1}})\cdot J$$

For the top shell, the metal plate induces negative charges on the upper surface in a positive electric field, and the intensity of the induced charge is related to the electric field intensity and the outside dielectric constant:

(11) $$\sigma =\varepsilon_{0}E_{0}$$

The ion charges can reach the top shell directly, and when the surface synthetic electric field on the middle interface reaches zero, the accumulated charge stops increasing.

By contrast, on the bottom shell, accumulated charges are redistributed over the whole device. The principle of superposition of charge surface density was used to balance the conductor to analyse the charge on the bottom shell.

In the absence of ion flow and when the uncharged device is placed into an electric field, the induced charge intensity of the top and bottom are represented by δ− and δ+:

(12) $$\delta +=\delta -=\varepsilon_{0}\cdot E_{0}$$

When no external electric field exists other than ion flow, the induced charge intensity of the top and bottom are the same $\delta^{\prime }$.

Combining the two situations, i.e., when the device is placed in an electric field, the accumulated charge of the top shell is zero, $\delta^{\prime }=\delta -$. Thus, the accumulated charge on the bottom shell is 2δ+.

### 3.3. Modulation of a Sensor in an Ion Flow Field

To determine the relationship between sensor detection and the original synthetic electric field strength, we constructed a simulation model. The sensor is encapsulated in a cube, which is a symmetrical structure. The top and bottom plate of the EFS is made of gold, and the sidewall is made of SiO_2_. We used a scale model to simulate the real situation. The EFS is 10 cm long and 10 cm wide. The background electric field is generated by a 120 cm long and 100 cm wide rectangle. Boundary 1 was set to a high voltage of 15 kV, and boundary 2 was set to ground potential. Thus, the background electric field was 15 kV/m. According to [24], the space charge density *ρ* over the entire region was set to 6 ×10^−7^ C/m^3^, except for inside the area of the sensor. The initial descent rate of the charged particles was 1 m/s. Additionally, the ion flow density was set to 100 nA/m^2^ based on previous experience with HVDC transmission lines [24]. Ion mobility ranges from 1.01 × 10^−4^ to 2.42 × 10^−4^ m^2^/(V∙s) under atmospheric conditions [24]; we used a constant ion mobility of 1.2 × 10^−4^ m^2^/(V∙s).

Under the following assumptions, we performed several simulations to study the influence of ion flow on the MEMS EFSs:

(1) The charge density around the sensor is set to a determined value.
(2) The charged particles will move along the electric field lines in the electric field. With time, the particles accumulated on the surface of the package of the sensor will generate a reverse electric field to stop particles of the same polarity from falling on the surface. We assume the charging process completes when no additional particles reach the metal shell of the sensor.

Transmission systems with different voltage levels cause different degrees of corona discharge. Because of the difference in ambient temperature and humidity, as well as the different operating modes of the transmission system, the space charge density will differ. Directly studying the influence of the space charge density on the MEMS EFS should offer an effective solution.

Charged particles in the electric field will undergo a complex movement process of collision and decomposition. The movement process is mainly influenced by the electric field force.

According to Gauss’s theorem, when the initial space charge density is zero, the surface charge density at the top and bottom surfaces induced under a 15 kV/m background electric field should be:

*e* = *ε*⋅*E* = 1.3275 × 10^−^^7^ (C/m^2^) (13)

The charge induced at the top surface is negative, and the charge induced at the bottom surface is positive. During the initial steps, the positive charge at the top surface will neutralize the initial induced negative charge until the charge density is zero. The accumulation of positive charge on the top surface will generate a reverse electric field to prevent ion flow. When the charging process is completed, the charge density of the top surface will not change.

**Figure 5:**
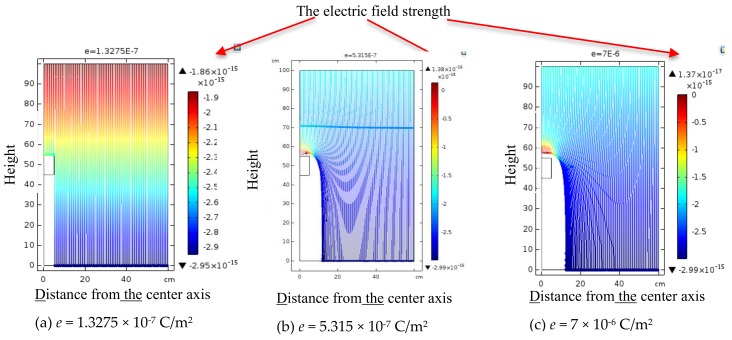
Particle trajectory of the initial state of top plate charged with different *e*.

The particle trajectories are shown in Figure 5, where the abscissa and ordinate represent the R-axis and Z-axis, respectively, and the colour indicates the electric field strength. Because the reverse electric field generated by the charge accumulated on the top surface is equal to the initial background electric field, charges with an initial velocity of 1 m/s remain on the top surface.

Figure 5 shows that when the charge density of *e* is increased, the electric field component of the R-axis increases, leading to the lateral offset of the ions. This phenomenon will becomes more apparent with increasing charge density on the top surface.

When charged particles are near the metal shell, a reverse electric field is generated by the positive charges on the top surface, which decreases the velocity of charged particles in the vertical direction to zero and increases the velocity of charged particles in the reverse direction. Additionally, the charged particles will move laterally due to the lateral imbalance in the electric field force generated by the positive charges on the top surface. Eventually, all charged particles will migrate around the sensor and move down, forming a charged air zone. The area of zero-charge density is called the ‘charge-empty area’.

### 3.4. Field Superposition Model

According to the previous simulation analysis, the top plate of the EFS can accumulate charged particles in the space electric field, forming a charge-empty area in the EFS adjacent area.

The sensor detection *E_in_* comprises three components: the original space electric field *E*_0_, the electric field *E*_1_ generated by the charged particles accumulated on the top surface, and the electric field *E*_2_ formed by the formation of the empty area, which is changed by the original space charge. We thus obtain the following equation:

(14) $$E_{in}=E_{0}+E_{1}+E_{2}$$

The sensor is sensitive only to the electric field in the vertical direction. Thus, the electric field in the other direction is not considered in this research. 

**Figure 6:**
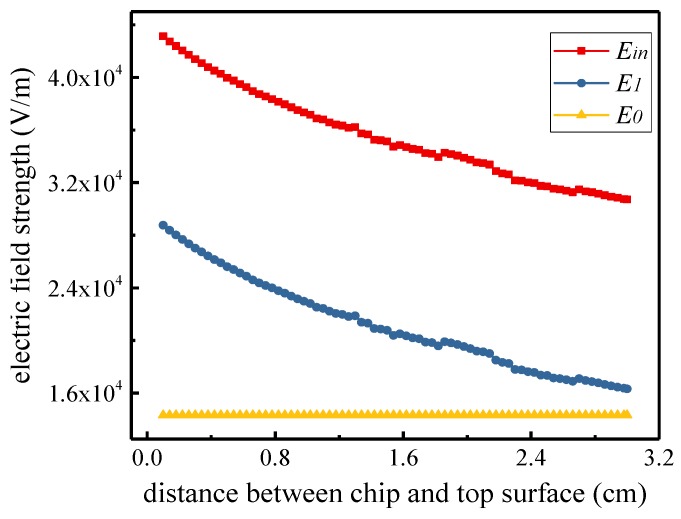
The modulation measurement results of *E_in_, E*_0_, and *E*_1_ at different distances between the sensor chip and the top surface.

We measured the value of each component by simulation to find the relationship among the components. First, we used parallel plates to generate an electrostatic field to simulate the electrostatic field generated by high-voltage transmission lines. In addition, the uniformly distributed space charges simulate ion flow near the transmission line. We measured *E*_0_ at different distances between the sensor chip and the top surface with the same boundary conditions described in part C of this section. We measured the electric field *E_in_* of the chamber in the simulation described in part C of this section. Finally, we used the same measurement method to measure the electric field *E*_1_ in the case of accumulating charge on the surface of the package. The results are shown in Figure 6.

Figure 6 shows that *E*_0_ plus *E*_1_ is approximately equal to *E_in_*. Thus, we ignored the component of *E*_2_. With further research, we found that *E*_1_ is proportional to the distance between the chip and the top surface. According to this relationship between *E*_1_ and the distance between the chip and top surface, we designed a differential EFS. The concrete structure is shown in Figure 1.

As shown in Figure 4, the two chips were placed at different heights. *E*_1_ is proportional to the distance between the chip and top surface, *E*_21_ = *λE*_11_, and *λ* is related to the location of the two chips.

From the following three equations:

$$E_{in1}=E_{0}+E_{11}$$

$$E_{in2}=E_{0}+E_{21}$$

(15) $$E_{21}=\lambda E_{11}$$

We obtained:

(16) $$\lambda =\frac{E_{in2}-E_{0}}{E_{in1}-E_{0}}$$

After *λ* is been determined, *E*_0_ can be obtained from *E_in_*_1_ and *E_in_*_1_:

(17) $$E_{0}=\frac{\lambda \cdot E_{in1}-E_{in2}}{\lambda -1}$$

As charge accumulates on the surface of the package, introducing charge into the earth is not necessary, thereby enabling potential independence. Using the upper shell of a package to accumulate the space charge, we measured the electric field in the ion flow. The difference structure was used to eliminate the influence of the large component *E*_1_, ensuring the accuracy of the measurement. Because of these characteristics, the sensor can measure the space synthetic electric field in an ion flow field.

## 4. Calibration and Measurement in an Electric Ion Flow Field

The ultimate goal of the present EFS is to measure the space synthesis electric field under HVDC transmission lines. First, we measured the zero drift of the EFS under zero field. Then, the relation coefficient between the output voltage of the EFS and the electric field to be measured is determined according to the output voltage of the EFS in a static electric field. After that, the calibration of the EFS in the ion flow field was thus completed. Finally, the EFS can be used to measure the synthetic electric field in an ion flow.

### 4.1. Introduction of the Parallel-Plate Ion Flow Generator

**Figure 7:**
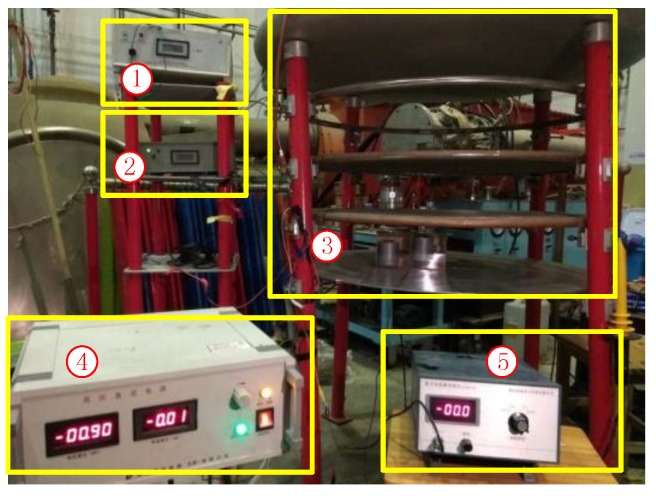
The measurement system in the laboratory. ① Corona voltage power supply (*Vc*); ② control voltage VA power supply (*Va*); ③ parallel-plate ion flow generator; ④ electrostatic field voltage power supply (*Vt*); and ⑤ a microgalvanometer.

To verify the function of the EFS and calibrate the coefficient of *λ*, we designed the measurement system shown in Figure 7. The structure of the parallel-plate ion flow generator was originally proposed by Withers et al. [25], and the equipment described in Ref. [25] (Figure 7) was used in the present work. 

The parallel-plate ion flow generator is composed of five flat plate layers with a diameter of 1 m on four insulated plastic columns. The circumjacent upward bending of the disc is designed to enable the generation of a uniform electric field around the disc and prevent discharge of the surrounding plates. The second layer is a Constantan wire net with a diameter of 0.3 mm and a length of two lines of 5 cm, which can produce a uniform ion flow. The third level is the control panel, which is made of a 0.8 cm × 0.8 cm stainless steel plate and acts as the first layer of the filter network. This layer enables a uniform distribution of corona ion flow. The voltage *Va* controls the intensity of the corona ion flow into the bottom parallel-plate electric field. The fourth layer is the upper plate of the parallel-plate electrode structure with a diameter of 0.142 mm and a length of two lines of 1.6 cm; it also serves as the second layer of the filter network. The fifth level is the lower plate of the parallel-plate electrode structure, which is a thick stainless steel ground plate. A hollow disc is positioned near the centre of the fifth level, which is used for the placement of the field strength tester. The uniform electric field in the ion flow field between the upper plate and the lower plate is key to this experimental device.

**Figure 8:**
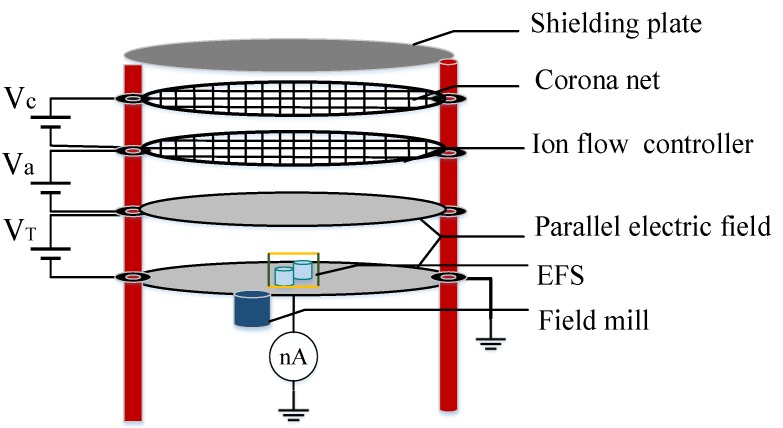
The structure of the ion flow generator.

As shown in Figure 8, when a high-voltage DC power was applied between the second or third layer, the corona phenomenon occurred around the thin copper wire, resulting in ion flow. By controlling the voltage, we controlled the space ion flow between the four or five layers. The EFS was placed in the middle of the fifth plate for measurement of the synthetic electric field in an ion flow. The microcurrent and field meters served as reference devices.

### 4.2. Measurements of Electrostatic Fields

**Figure 9:**
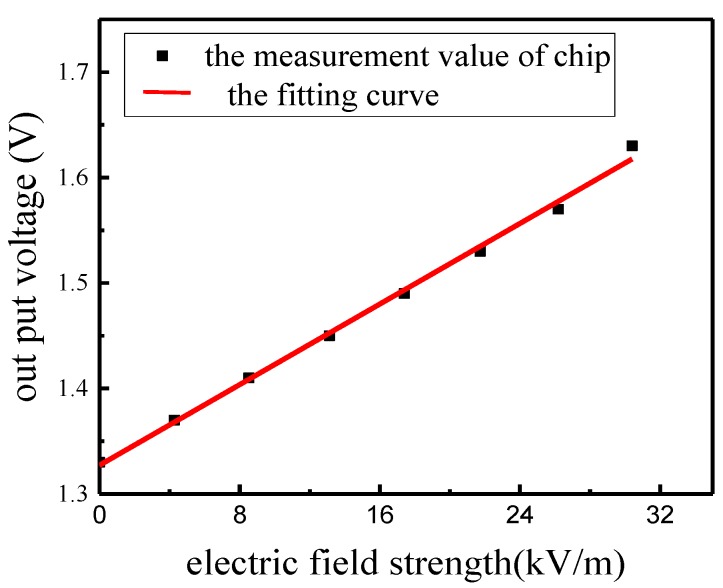
The measurement results in an electrostatic field.

We first tested and verified the function of the EFS in an electrostatic field, which required determining the zero drift of the EFS in the zero field and identifying the coefficient between the output voltage and the electric field. In this section, we utilized a DC voltage *Vt* and the parallel plates in Figure 8 to generate an electrostatic field. The EFS was placed in the middle of the fifth plate to measure the electric field strength at different *Vt*. The measurement results are shown in Figure 9.

As shown in Figure 9, the zero drift of the EFS under the zero-field condition was 1.36 V and the relation was highly linear. Thus, the sensor was shown to be an ideal device for measuring an electric field.

### 4.3. Calibration of the Electric Field in an Ion Flow Field

Consistent with the aforementioned discussion, the measurement results of the MEMS sensor chip in the package include three parts. The use of a double cylindrical composite potential independent package structure enabled indirect measurement of the original electric field strength *E*_0_, eliminating the interference of *E*_1_, which is the relatively larger contributor to the electric field distortion. The parameter *λ* is related only to the relative height of the two chips in the encapsulation structure and is independent of the applied electric field. Thus, this parameter can be calibrated according to Equation (16) after the package structure has been determined. The principle of calibration of the EFS in an ion flow field is described in great detail in the following paragraphs.

Due to the distortion of the electric field, demarcating and calibrating the EFS is difficult, and measurements without demarcation and calibration processes are meaningless. A small part of the EFS is calibrated in an electric field [25]. Although the authors of Ref. [25] used a parallel-plate ion current generator to calibrate their EFS, the value measured by the field mill on the ground was not equal to the original value of the space synthetic electric field. The authors of a previous study [26] proposed a method for artificially manufacturing an electric ion flow field, which is the approach recommended by IEEE for DC ion flow field measurements and calibration, and a calibration method for an electric field in space. However, these authors [26] mistakenly calculated the *E_d_* to be zero [27]. Because *J* = *pkE*, where *p* is the space charge density and *J* should be a constant along *z* due to the continuity of current, if *E_d_* = 0, *J* at anywhere in the measurement region would be 0 [27]. In the present study, we propose a calibration method for a space synthetic electric field measurement in space using a parallel-plate ion current generator, microcurrent meter, field mill and several DC power supplies. 

The space is filled with ions due to corona discharge. The function of the electric field at each point should satisfy the Poisson equation [26]:

(18) $$\nabla \cdot \vec{E}=\rho /\varepsilon$$

The ions formed by corona discharge move with the electric power line under the action of the electric field, thus forming an ion current. The ion current density *J* can be described as *J* = *kpE*, where *k* is the ion flow mobility*, p* is the density of space charges and *E* is the synthetic electric field. According to the Poisson equation and the relationship between *J* and *k*, s, *p* and *E*, we obtained the following equation [26]:

(19) $$E(z)={\left(E_{n}^{2}-\frac{2Jz}{k\varepsilon_{0}}\right)}^{1/2}$$

The fifth layer is the zero point of the Z-axis, and the direction is vertical. *E_n_* is the electric field on plane of the fifth layer. By integrating Equation (19), we obtain the voltage equation:

(20) $$V(z)=V_{T}+(\frac{k\varepsilon_{0}}{3J})[{\left(E_{n}^{2}+\frac{2J(z-d)}{k\varepsilon_{0}}\right)}^{3/2}-E_{n}^{3}]$$

where *V_T_* is the voltage applied to the fourth layer plate. Equation (20) satisfies *V*(0) = 0 because the fifth layer plate is grounded. Thus, we obtain:

(21) $${\left(E_{n}^{3}-\frac{3JV_{T}}{k\varepsilon_{0}}\right)}^{2}={\left(E_{n}^{3}-\frac{2Jd}{k\varepsilon_{0}}\right)}^{3}$$

By solving Equation (21), we obtain the mobility of the ion flow:

(22) $$k=\frac{-B+\sqrt{B^{2}-4AC}}{2A}$$

$$A=6(V_{T}-E_{0}d)JE_{n}^{3}$$

$$B=(9V_{T}^{2}-12d^{2}E_{n}^{2})J^{2}\varepsilon_{0}$$

$$C=8J^{3}d^{3}$$

The ionic current mobility *k* is included in Equation (22), and the intensity of the electric field in the air can be obtained:

(23) $$E(z)={\left(E_{n}^{2}+\frac{4AJz}{\varepsilon_{0}\frac{-B+\sqrt{B^{2}-4AC}}{2A}}\right)}^{1/2}$$

The fourth layer plate voltage *V*_*T*_ is known. The current density *J* can be measured by the microcurrent meter. Furthermore, the ground electric field *E_n_* can be measured by the field mill. Because the parameters of *V*_*T*_, *J* and *E_n_* can be obtained, according to Equation (23), the air synthetic electric field in space induced by ion flow can be calculated. The parameter *λ* can then be obtained by Equation (16). Finally, the EFS can be used to measure the unknown space synthetic electric field in ion flow field according to Equation (17).

The encapsulation sidewall shell used in this calibration is made of Teflon material. The height of chip1 is 0.6 cm from the top of the shell, and the height of chip 2 is 2.3 cm from the top of the sensor. The distance between the second level and third level is 1 dm. We set the voltage *Vc* to 2.5 kV and adjusted the voltage *Va*. To realize the measurement of the synthetic electric field of ion flow field, the sensor demarcation is needed. The demarcation results is shown in Table 3. According to the aforementioned method, parameter *λ* is displayed in the following table.

Substituting the data shown in Table 3 into Equation (16), we obtained the demarcated value of *λ* = 1.861. According to Equation (17), because parameter *λ* has been determined, the unknown synthetic electric field in space or on ground and in an electrostatic field or in an ion flow field can be measured using the differential EFS we designed.

**Figure 10:**
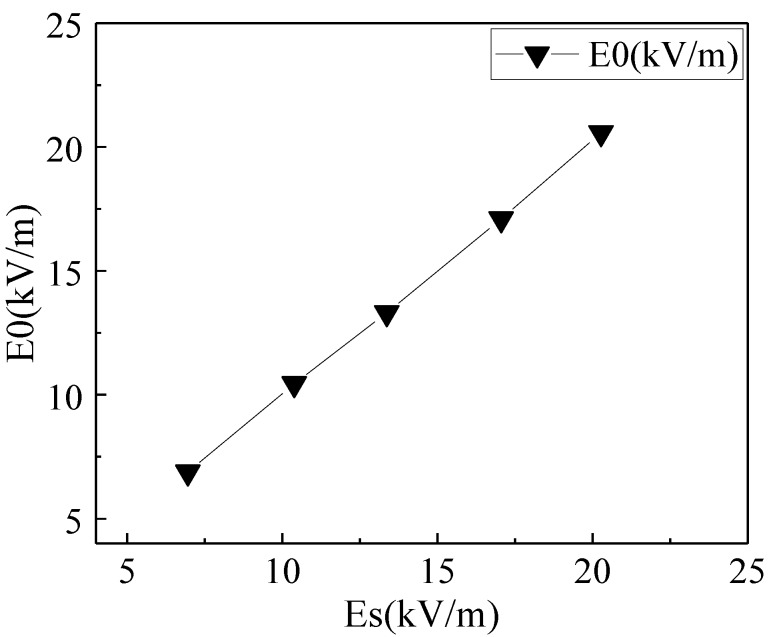
Comparison of the measured values *E*_0_ and the calculated values *Es*.

To verify the performance of the double differential EFS, we conducted a series of calibration experiments. Under the same experimental conditions, the double differential EFS measured the synthetic electric field induced by ion flow, which was generated and adjusted via the intensity of the ionic current and the electrostatic field. Additionally, the theoretical value of *Es* was obtained by a field mill according to Equation (22). The performance of the sensor was evaluated by comparing the measured values of *E*_0_ and the calculated values of *Es*. A comparison of the results is given in Figure 10.

Through this demarcated value of *λ*, the measured values of the ion-current-space electric field under different conditions were obtained. As shown in Figure 10, the measured values are consistent with the calculated results, which indicates that the differential EFS proposed in the present study shows good linearity and accuracy. The experiments show that the method is practical and effective.

## 5. Conclusions

To measure the space synthetic electric field in an ion flow field, we designed a double potential independent EFS based on MEMS technology. Compared with other EFSs, the present sensor has the advantages of small volume, independent potential (without grounding), and the ability to support the measurement synthetic electric fields in space.

We first designed an electrostatic comb-driving and single-layer sidewall induction EFS. The theory of the driving and the induction components were analysed. We then designed the package of our sensor. The upper and lower plates were made of metal, and the sidewalls were made of an insulating material. When the EFS was placed in an ion flow field, the upper plate accumulated charged articles, distorting the original electric field. Through a multi-physical field simulation, we found a ‘charge-empty area’, and analysed the measurements, which consisted of three components: the original field *E*_0_, the additional electric field *E*_1_ caused by ion flow in the area, and the electric field distortion *E*_2_*.* Because the component *E*_2_ is small compared with the other two fields, we ignored the influence of *E*_2_. To eliminate the effect of *E*_1_, we designed a differential potential independent structure.

To better characterize the performance of the EFS, a parallel-plate ion flow generator was designed, analysed, fabricated and then used to generate an ion flow for the EFS measurements. Due to the distortion of the electric field, demarcating and calibrating the EFS was difficult. A calibration method for an ion flow electric field in space was proposed based on the ion flow generator.

In the ion flow field demarcation experiments, the measured value of λ was 1.861. Comparison of the simulation results with the theoretical values revealed that the proposed EFS shows good linearity and accuracy. Simulation and experimental results showed that the present potential independent differential structure exhibits good performance for measuring an ionized electric field.

## Figures and Tables

**Table 1:** Comparisons of electrical field sensor.

Properties	Field Mill [7,8]	MEMS Sensor [14]	Distortion-Free Probe [16]	In This Paper Differential EFS
Schematic Diagram	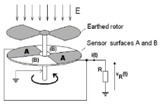	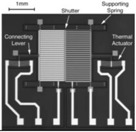	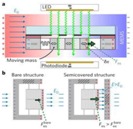	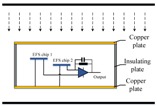
Distortion	large	small	small	large
Measures of ion flow	Yes	No	No	Yes
Grounding	Yes	No	No	No
Cost	low	low	high	low
Spatial electric filed in ion flow measurement	No	No	No	Yes

**Table 2:** The parameters of the EFS.

The width of the shielding electrode	10 μm	*a* (as shown in Figure 3)	5 μm
The length of the shielding electrode	900 μm	*d* (as shown in Figure 3)	12 μm
The width of the sensing electrode	10 μm	*g* (as shown in Figure 3)	5 μm
The width of the sensing electrode	900 μm	The number of driving electrodes	250
The gap between the adjacent sensing and shielding electrodes	15 μm	The thickness of the device layer	20 μm
The number of sensing electrodes	50		

**Table 3:** Demarcation experiment of *λ* in an ion flow field.

*V_T_* (kV)	*I* (nA)	*E_n_* (kV/m)	*Es* (kV/m)	*E_in_*_1_ (kV/m)	*E_in_*_2_ (kV/m)	λ
0.63	1.3	5.31	3.478	8.66	6.56	1.861
